# Attitudes of mental health providers towards adoption of evidence-based interventions: relationship to workplace, staff roles and social and psychological factors at work

**DOI:** 10.1186/s12913-019-3933-4

**Published:** 2019-02-08

**Authors:** Marte Rye, Oddgeir Friborg, Ingunn Skre

**Affiliations:** 1 Department of Psychology, UiT The Arctic University of Norway, N-9037 Tromsø, Norway; 2 Department of General Psychiatry and Addiction, University Hospital of North Norway, N-9038 Tromsø, Norway

**Keywords:** Evidence-based interventions, Implementation, Attitudes, Therapists, Mental health

## Abstract

**Background:**

Gaining insight into factors influencing the adoption of evidence-based interventions (EBI) is essential to ensuring their sustainability in the mental healthcare setting. This article describes 1) differences between professional staff roles in attitudes towards EBI and 2) individual and organizational predictors of attitudes towards adopting EBI.

**Methods:**

The participants were psychologists and psychiatric nurses (*N* = 792). Student *t*-tests were used to investigate group differences of global attitude scores on the Evidence-based Practice Attitude Scale-36 (EBPAS-36). A confirmatory factor analysis (CFA) of the EBPAS-36 measurement model, and a principal component analysis (PCA) of the factor scores were used to obtain attitudinal components for the subsequent hierarchical regression analyses.

**Results:**

Three second-order attitudinal components were retained and named: *professional concern*, *attitudes related to work conditions and requirements*, and *attitudes related to fit and preferences*. Nurses’ global attitudinal scores were more positive than those of psychologists, while clinicians had less positive global attitudinal scores than non-clinicians. Hierarchical regression analysis showed that provider demographic, social and psychological factors in the workplace and staff role predicted attitudes towards adopting EBI, e.g. male gender, older age and working in private practice predicted more negative global attitudes, while working in academia, experiencing social support from colleagues and empowering leadership predicted more positive global attitudes to adopt EBI. The prediction outcomes for the specific attitudinal components are presented, as well.

**Conclusion:**

The findings suggest that implementation efforts may benefit from being tailored to the different needs and values of the affected professionals, including the role of the context they operate within. Implications with a special emphasis on training efforts and organizational development are discussed.

## Background

Worldwide, significant resources are allocated to the development and application of evidence-based treatment programmes and interventions (EBI) in mental health care. However, interventions that show strong empirical support are infrequently implemented in real-life clinical service settings and often fail to cause the expected change in practice [1–5]. The increasing realization from implementation science is that understanding the factors underpinning the actual willingness and decision to adopt an intervention, is necessary in order to proceed with a successful implementation [6]. The adoption or the earliest phase(s) of the implementation process represents a period where decisions to continue (or not) with a full implementation are affected, as well as how the implementation should be done. Consequently, challenges in the early phase of implementation may substantially impact the subsequent implementation process by, e.g., hampering sustainability within the service setting or even lead to a de-implementation [6]. Both individual and organizational factors play a major role in implementation processes, but more knowledge is needed to understand the interplay between these factors [7–9]. Gaining insight into the role of individual professional provider characteristics and organizational context factors may provide a better understanding of how to overcome adoption obstacles; thus, helping to tailor implementation strategies in order to anchor the implementation and increase the uptake of EBI.

### Individual and organizational implementation factors

The attitudes of individual mental health professionals are central to the adoption and use of EBI [6, 8, 10, 11], since attitudes towards change and innovation may shape the initial decision process, as well as intentions to try new practices [8, 12]. Moreover, the attitudes of mental health professionals are often mixed, including both enthusiasm and ambivalence towards EBI [11]. Attitudes of professionals also influence and interact with a number of individual demographic factors (e.g., gender, years of experience) and organizational factors (e.g., leadership, social climate and organizational support, policies and system factors) [6–8, 10, 13–18]. For instance, females have reported experiencing *less* time and administrative burdens in connection with learning EBI, compared to males [8], while providers with higher caseloads have reported *greater* time and administrative burdens [8]. Additionally, more experienced clinicians have reported a greater perception of therapy as a balance between art and science [8], whereas less experienced clinicians have reported a greater openness to new practices [19] and valued job security and organizational support for learning new EBI [16]. A survey assessing barriers to and facilitators of adopting EBI among psychotherapists pointed to training issues (e.g., insufficient time, a high cost and lack of training), clinicians’ attitudes (e.g., concerns with a new technique’s efficacy, beliefs that the current practice was sufficient and that treatment must be compatible and easy to integrate with the existing therapeutic approach), as well as contextual and institutional factors (e.g., a lack of administrative support, and heavy caseloads) [17]. Also from the somatic health sector, Norwegian nurses pointed to time demands and a lack of skills in locating, understanding and implementing research findings into routine practice as substantial barriers [20]. Practical and logistic factors, such as limited time, high costs and lack of resources, stand out as barriers across several studies from the mental health field [21–24]. As facilitators, higher levels of positive leadership styles, proactive implementation leadership, engaged organizational culture and a climate characterized by high levels of educational support and consultations are associated with more positive provider attitudes to adopting interventions [7, 25, 26]. Conversely, poor organizational climate is associated with a greater perceived divergence between practice as usual and the adoption of EBI [27].

### Differences between various professional roles and positions

While some studies have observed no differences among professional disciplines in attitudes to adopting EBI [28], other studies have observed that individuals trained in social work scored higher on global attitudes to adopting EBI and openness to new practices than those trained in psychology [19]. A qualitative cross-disciplinary study of barriers to and facilitators of evidence-based practice among dental hygienists, nurses and psychiatrists reported that psychiatrists expressed a greater mistrust of research publications, while dental hygienists and nurses reported having to negotiate with superiors to introduce changes [29].

Most studies of mental health care provider attitudes have sampled therapists, omitting other relevant stakeholders. This is unfortunate, as other stakeholders may have concerns or attitudes that deviate from those of therapists [30–32]. For instance, a study comparing policy makers to stakeholders (clinicians, administrative staff and consumers) involved in practice revealed that the practice group ascribed a greater importance to the impact of implementation on clinical practice, for instance, expressing concerns with how new interventions might impact the therapeutic relationship and possibilities for individualizing treatment [32]. Differences between leaders and therapists also emerge; one study observed different preferences for specific EBI, hypothesized by the authors to reflect leaders and therapists having different priorities and values, e.g., concerning organizational investment (costs, training resources and staffing) and end-user experiences such as the ease of use and clinical utility [31]. As leadership also plays an important role in implementation processes, for instance, through fostering an organizational climate facilitating implementation processes and allocation of resources [31, 33–35], the perspective of these staff roles should be of equal interest.

### Aims

The current article explored organizational and individual factors related to attitudes towards implementation processes and adoption of evidence-based interventions. More specifically, the aims were to:

1. Investigate the differences among professional staff roles in attitudes to adopt EBI, e.g., psychologists vs. nurses, clinicians vs. non-clinicians, and leaders vs. non-leaders.
2. Identify provider demographic and organizational predictors of attitudes to adopting EBI.

To achieve this, the Evidence-based Practice Attitude Scale-36 (EBPAS-36) was used to measure providers’ attitudes to adopt new interventions, while The Nordic Questionnaire for Psychological and Social Factors at Work (QPS Nordic) was used to explore social work climate and organizational predictors. Based on the literature, we hypothesized that more positive organizational predictors (i.e., social support, encouraging and developing leadership) would predict more positive global and domain specific attitudes to adopting EBI, while the presence of greater job demands would be associated with more negative or ambivalent attitudes. Further, given the exploratory nature of the study, we hypothesized that both provider demographic and organizational predictors and staff roles would be differentially associated with underlying attitudinal domains, contributing to the developing literature on how implementation strategies need to be targeted to secure adherence to interventions in everyday practice.

## Methods

### Procedure and sample

The overall procedure is described elsewhere [9]. Participants were members of the Norwegian Psychological Association (Sample 1, *N =* 3598) and the Norwegian Nurses Organization, professional group for nurses in mental health and substance abuse (Sample 2, *N* = 1436). Participants were invited by emails sent out by their respective organizations, containing information about the study as well as a web link providing access to the survey.

A total of 856 psychologists and psychology students (24.0% response rate for sample 1) and 191 nurses (13.3% response rate for sample 2) completed the survey (*N* = 1047). For the present study, subjects not completing any of the EBPAS-36 items and those with missing data on entire EBPAS-36 subscales were excluded (*n* = 192). Since the aim of the paper concerned attitudes of professional practitioners and their work settings, the psychology students (*n* = 63) were excluded from the sample. Thus, the final sample (*N* = 792) included 671 licensed psychologists (84.7%) and 121 licenced nurses (15.3%).

### Measures and assessment

#### Conceptualization

The survey was framed within the context of “specific research-supported interventions only” (i.e., therapies and methods) [9]. This was done in an effort to make the important distinction between the more comprehensive concept of *evidence-based practice (EBP)*, defined as *“the integration of the best available research with clinical expertise in the context of patient characteristics, culture, and preferences”* and *evidence-based interventions (EBI)*, referring to specific research-supported interventions [36, 37]. It has been assumed that a confusion or misinterpretation of these concepts may play a role in providers’ ambivalent perceptions of evidence and research findings per se, as well as the integration of science into routine practice [11].

#### Demographic variables

The demographic variables included gender, age, educational level and workplace (all using response categories as shown in Table 1), as well as the number of years worked in substance abuse and/or mental health service, having leadership responsibilities (yes/no), working as a clinician (yes/no), and professional discipline (psychologists/nurses).

**Table 1:** Demographic characteristics

Characteristics	Psychologists		Nurses	
	(*n* = 671)		(*n* = 121)	
Gender				
Female	428	(63.8)	102	(84.3)
Male	227	(33.8)	15	(12.4)
Missing	16	(2.4)	4	(3.3)
Age				
≤ 30 years	70	(10.4)	2	(1.7)
31-40 years	239	(35.6)	17	(14.0)
41-50 years	167	(24.9)	30	(24.8)
51-60 years	110	(16.4)	53	(43.8)
≥ 61 years	82	(12.2)	19	(15.7)
Missing	3	(0.4)	0	(0)
Highest Education - Clinical Psychologists^a^				
Both Ph.D. and clinical specialist degrees	28	(4.2)	n/a	n/a
Ph.D.	26	(3.9)	n/a	n/a
Clinical specialist degree	322	(48.0)	n/a	n/a
Other continued education	23	(3.4)	n/a	n/a
Missing	2	(0.3)	n/a	n/a
Highest Education - Nurses^b^				
Ph.D.	n/a	n/a	2	(1.7)
Master’s degree	n/a	n/a	18	(14.9)
Other continued education	n/a	n/a	97	(80.2)
Missing	n/a	n/a	2	(1.7)
Working as clinicians	586	(87.3)	105	(86.8)
Tenure in substance abuse and mental health (years)	10.4	(9.9)	18.0	(9.2)
Managerial responsibilities	160	(23.8)	19	(15.7)
Working evidence-based	346	(51.6)	78	(64.5)
Type of workplace				
Outpatient units - adults	119	(17.7)	20	(16.5)
Outpatient units - youth	71	(10.6)	2	(1.7)
Outpatient unit - abuse	30	(4.5)	4	(3.3)
Inpatient unit >2 months	30	(4.5)	10	(8.3)
Inpatient unit <2 months	22	(3.3)	11	(9.1)
Research/education clinical	32	(4.8)	0	(0)
Research/education non-clinical	39	(5.8)	6	(5.0)
Private practitioners with subsidies^c^	49	(7.3)	n/a	n/a
Private practitioners without subsidies^d^	27	(4.0)	n/a	n/a
Governmental (e.g., family counselling services)	39	(5.8)	1	(0.8)
Municipal health and care services	70	(10.4)	32	(26.4)
Other^e^	141	(21.0)	29	(24.0)
Missing	2	(0.3)	6	(5.0)

Data presented as n (%) or mean (SD), if appropriate. n/a = categories not applicable. ^a^After an initial cand.psychol. degree. ^b^After an initial Bachelor degree. ^c^Psychologists with clinical specialist degree working in private practice with operating subsidies from the Norwegian state, meaning patients’ costs of treatment exceeds the covered costs of public help. ^d^Psychologists and nurses working in private practice without subsidies, see^c^. ^e^Clinicians with a combination of multiple work settings, e.g., both inpatient and outpatient patients

#### Attitudes

Attitudes were measured with the Evidence-based Practice Attitude Scale-36 (EBPAS-36) Norwegian version. The EBPAS-36 [9] is a short version of the Evidence-based Practice Attitude Scale-50 [8], validated in both US and Norwegian samples [8, 9]. The EBPAS-36 assesses mental health and social service provider’s attitudes towards adopting evidence-based interventions. While the original EBPAS instrument consisting of 15 items measured 4 attitudinal domains [19, 38], the subsequent work on the EBPAS-50 and EBPAS-36 expanded the instrument to be able to cover a wider domains of attitudes [8, 9]. For a full description and presentation of the EBPAS-36 and its items, we refer to Rye et al., 2017 [9]. The EBPAS-36 items cover 12 subscales, each with 3 items (the subscale names are provided in italics): 1) the likelihood of adopting EBI given *requirements* to do so (subscale Cronbach alpha for current sample, α = .92), 2) the intuitive *appeal* of adopting EBI (α = 0.60), 3) *openness* to new practices (α = 0.75), 4) the perceived *divergence* of providers usual practice from research-based or academically developed interventions (α = 0.70), 5) *limitations* of EBI and their inability to address client needs (α = 0.86), 6) EBI *fit* with the values and needs of the client and clinician (α = 0.59), 7) negative perceptions of *monitoring* (α = 0.84), 8) *balance* between perceptions of clinical skills and science (α = 0.67), 9) time and administrative *burden* of learning an EBI (α = 0.76), 10) *job security* related to using/learning an EBI (α = 0.85), 11) perceived *organizational support* for adoption (α = 0.86), and 12) positive perceptions of receiving *feedback* (α = 0.83). The total score represents a respondent’s global attitude towards EBI (α = 0.87). The items are formulated as statements, and responses are given on a 5-point Likert scale (from 0 designating “not at all” to 4 meaning “to a very great extent”). To reduce response biases, 15 items belonging to five subscales (divergence, limitations, monitoring, balance and burden) are negatively framed and reverse-scored before computing the total score. A higher total score indicates a more positive global attitude to adopting EBI. The EBPAS-36 has shown adequate psychometric properties with regard to reliability, construct- and cross-cultural validity and being pragmatic [9].

#### Organizational features and work climate

Organizational features and work climate was measured with The Nordic Questionnaire for Psychological and Social Factors at Work (QPS Nordic). The QPS Nordic is developed from organizational theories [39]. The instrument consists of 129 items assessing work-related tasks and individual, social and organizational factors. For the present study, a selected set of subscales was chosen following discussion with colleagues regarding relevant subscales. Based on their feedback, a consensus discussion among two of the authors (MR and IS) led to the inclusion of the following six subscales (20 items) as most relevant for the study aims: 1) *quantitative job demands* (4 items, subscale Cronbach alpha for current sample, α = .83) measuring the extent of the experienced workload, 2) *control over decisions* (5 items, α = 0.75) measuring the influence on decisions regarding own work place, workload, work methods and collaborating partners, 3) *support from colleagues* (2 items, α = 0.80) asking for an assessment of social interaction when needing collegial assistance, 4) *support from the nearest superior* (3 items, α = 0.91) measuring the social interaction when needing a superior’s assistance, 5) *empowering leadership* (3 items, α = 0.90) assessing encouragement from superiors in decision-making, sharing personal opinions and development of abilities, and 6) *social climate* (3 items, α = 0.73) measuring the social climate at the workplace. In addition, 1 single item from the domain organizational climate was used: “What is the climate like in your work unit? Rigid and rule-based”. Responses are given on a 5-point Likert scale ranging from 1 representing “very little or not at all” to 5 “very much”, or 1 “very seldom or never” to 5 “very often or always”, as appropriate. As for the whole instrument, the QPS has shown acceptable psychometric properties [40]. The predictive validity related to long-term sick leave is good [41]; hence, comparisons between professional groups are valid [40, 41].

### Statistical analyses

#### *Confirmatory factor analysis* (CFA)

To develop a second-order model of attitudes towards adopting EBI to use as outcome variables for predictive analysis, a CFA was conducted in Mplus v8. The model specification was based on the 12 subscales of the recently developed EBPAS-36. The parameters were estimated with the full maximum likelihood estimation procedure (FIML). Robust standard errors (MLR) were obtained to accommodate non-normal item distributions. The following model fit indices were used: χ^2^, root mean square error of approximation (RMSEA), standardized root mean error (SRMR) and comparative fit indices (CFI). In line with Hu and Bentlerʼs cutoff recommendations [42], RMSEA values < .06, SRMR < .08 and CFI > 0.95 indicate an acceptable model fit. The primary factor scores were saved in Mplus and subjected to an exploratory second-order principal component analysis (PCA), using SPSS v25. Correlation analysis, t-tests and hierarchical regression analysis were conducted in SPSS v25. Missing EBPAS-36 and QPSnordic item scores were imputed using the Expectation Maximization (EM) method. Values were imputed separately for each subscale’s set of items. Bivariate associations were calculated as Pearson correlation coefficients.

#### Multiple regression analyses

Hierarchical multiple regressions models were built to examine the predictive value of demographic background variables, and social and psychological factors at work for attitudes towards adopting EBI. Categorical predictors with three or more categories were dummy coded. Data were checked for influential cases according to Cook’s distance criteria, with no values with Cook’s distance greater than 1. For all analyses, predictor variables were entered in the same predefined blocks. Within each block, variables not contributing to the prediction were manually removed. In the first block, gender, age and years of experience were entered. The highest level of education was entered in the second block. In the third block, workplace and the indicator of being employed at a work site working systematically with one or more EBI were entered. In the fourth block, QPSnordic subscales and the single QPSnordic item regarding the social climate being rule-based and rigid were entered. Indicator variables of the staff role being a clinician, holding a position of a psychologist or a nurse and having leadership responsibilities were in the fifth and last block. The order of entering variables was decided to examine whether staff role contributed significantly to the model after controlling for all other predictor variables, including QPSnordic variables.

## Results

Descriptive data of the two samples are given in Table 1. Both samples were made up mostly of women. Nurses were older and reported more years of clinical experience. The majority in both samples worked as clinicians. Comparable proportions of psychologists and nurses held managerial responsibilities.

### Second-order model of attitudes

The CFA of the 12 EBPAS-36 subscales yielded acceptable model fit indices, as indicated by a low degree of misspecification errors (RMSEA = .048 (CI_90%_ .045–.050); SRMR = .064) and a fair incremental fit (CFI = .92, TLI = .90). The CFA factor scores were saved in Mplus and subjected to a principal component analysis (PCA) in SPSS, hence representing a second-order factor analysis. The analysis extracted four components with eigenvalues greater than 1 (*R*^2^ = 74.7%). However, a simpler three component solution was preferred due to fewer cross-loadings and being more parsimonious. The component loadings were promax rotated and are presented in Table 2. The first component was labelled *professional concern*, as it included perceived limitations of EBI (subscale 5), balance and divergence between clinical practice and science (subscales 8 and 4), negative perceptions of monitoring (subscale 7), and a lack of openness to new practices (subscale 3). The second-order component was labelled *attitudes related to work conditions and requirements*, encompassing the time and administrative burdens of learning new interventions (subscale 9), job security and perceived organizational support (subscales 10 and 11) and adoption of imposed evidence-based interventions (subscale 1). Burden (subscale 9) had a high cross-loading on the *professional* c*oncern* component, thus indicating both second-order dimensions. The appeal subscale also had a significant cross-loading on the third second-order component labelled *attitudes related to fit and preferences*, thus also being partly explained by this factor. The third and last component reflected the personal willingness to use new interventions based on autonomy, fit with the values, preferences and needs of both patient and provider, as well as positive perceptions of feedback.

**Table 2:** Second-order principal components analysis and correlation among components (*N* = 792)

PCA components	Component loadings		
	1	2	3
Professional concerns			
Limitations	.83		
Divergence	.81	-.34	
Balance	.76		.48
Monitoring	.72		
Openness	-.60	.38	
Work conditions and requirements			
Burden	.67	.74	
Organizational support		.68	
Job security		.62	
Appeal		.62	.47
Requirements		.61	
Fit and feedback			
Fit			.84
Feedback			.68
Eigenvalues	4.39	2.37	1.19
Explained variance (%)	36.57	19.73	9.93
Correlations^a^			
Professional concern	--		
Work	-.30**	--	
Fit	-.08*	.30**	--

^a^Pearson’s r coefficients between second-order components. *Indicates significance at the *p* < .05 level, ** indicates significance at the *p* < .001 level

### Aim 1: Differences between different professional roles and positions

On the EBPAS-36 total scale, psychologists as a group reported lower global attitude scores (M = 2.67, SD = 0.47) than nurses (M = 2.76, SD = 0.39); this difference was statistically significant (95% CI, −.18, −.00), *t*(790) = − 2.06, *p* = .039). The effect size difference was small (*g* = 0.20). Respondents working as clinicians reported significantly lower global attitude scores (M = 2.67, SD = 0.47) than non-clinicians (M = 2.78, SD = 0.39); this difference was statistically significant (95% CI, −.20, −.01), *t*(790) = − 2.10, *p* = .036). This effect size difference was also small (*g* = 0.24). For leaders vs. non-leaders, the difference in EBPAS-36 total scale score was not significant (M = 2.72, SD = 0.46 and M = 2.68, SD = 0.46, respectively; *t*(780) = 1.02, *p* = .31).

The mean EBPAS-36 scores indicating global attitudes among the different professional roles and positions ranged between 2.67 and 2.78. Comparable mean scores for the EBPAS-36 measure, as used in the current study, is lacking. However, as compared to the study by Okamura et al., [16], the mean scores were all below their reported mean EBPAS-50 total score on 2.89, and comparable with mean EBPAS-15 total score on 2.73 from an examination of U.S. norms in a national U.S. sample of 1089 mental health providers across 26 states [19].

### Aim 2: Predictors of attitudes towards adopting EBI

The results of the regression analyses are presented in Table 3. The model predicting global attitudes to adopting EBI was statistically significant (*R*^2^ = .20, *F*(11, 744) = 17.01, *p* < .0005; adjusted *R*^2^ = .19). The variables contributing significantly to the full model were as follows: gender (males scoring lower than females) and age (older individuals scoring lower than younger) in block 1; workplace (individuals in non-clinical research and educational settings scoring higher, while private practitioners scored lower than the reference group) and working at a site systematically applying one or more EBI (scoring higher than working outside such sites) in block 3; social and psychological work factors (individuals receiving support from colleagues and experiencing empowering leadership scoring higher, while those reporting control over their decisions scored lower) in block 4. Staff role in block 5 did not contribute statistically to the model. The following effects dropped out as variables were added to the model: other continued education, having dual competence and being in a governmental workplace.

**Table 3:** Hierarchical Multiple Regression with factors predicting EBPAS-36 total score and second-order components

	EBPAS-36 total scale^a^			Professional concerns^b^			Work conditions and requirements^c^			Fit and preference^d^		
Step and predictor	∆ R^2^	Init. β	Fin. β	∆ R^2^	Init. β	Fin. β	∆ R^2^	Init. β	Fin. β	∆ R^2^	Init. β	Fin. β
Step 1:	.07**			.01*			.12**			.04**		
Gender, female (ref)		-.13**	-.10*		n.s			-.12**	n.s		-.18**	-.16**
Age		-.21**	-.15**		n.s			-.32**	-.24**		-.08*	n.s
Years of experience		n.s			.12**	.09*		n.s			n.s	
Step 2:	.01*			.02**			.01*			.01*		
Education, specialist/MA (ref)												
Cand.Psychol/Bachelor nurse		n.s			n.s			n.s			n.s	
Unfinished cont. education		n.s			n.s.			n.s			n.s	
Other		.11*	n.s		-.09*	n.s		.09*	n.s		n.s	
Ph.D.		.n.s			n.s			n.s			-.10*	-.09*
Dual competence		.08*	n.s		-.12**	-.07*		n.s			n.s	
Step 3:	.08**			.07**			.07**			.00		
Workplace, Outpatient (ref)												
Inpatient		n.s			n.s			n.s			n.s	
Research/education clinical		n.s			n.s			-.10*	-.10*		n.s	
Research/education non-clinical		.10*	.12**		-.10*	-.11*		n.s			n.s	
Private practitioners		-.19**	-.12*		.16**	.10*		-.24**	-.18**		n.s	
Governmental		.08*	n.s		-.08*	n.s		n.s			n.s	
Municipal		n.s			n.s			n.s			n.s	
Other		n.s			n.s			n.s			n.s	
Systematically evidence-based		.13**	.09*		-.13**	-.09*		-.07*	n.s		n.s	
Step 4:	.04**			.04**			.06**			.06**		
Social climate, rule-based		n.s			.10*	.10*		n.s			n.s	
Job demands		n.s			n.s			.19**	.19**		n.s	
Control decisions		-.09*	-.09*		.09*	.10*		-.13**	-.13**		.12**	.14**
Social climate		n.s			n.s			.09*	.09*		n.s	
Support colleagues		.13**	.13**		-.08*	-.08*		n.s			.13**	.13**
Support superiors		n.s			n.s			n.s			n.s	
Empowering leadership		.15**	.15**		-.13*	-.12*		n.s			.09*	.11*
Step 5:	.00			.01*			.00			.01*		
Working as clinicians		n.s			n.s			n.s			n.s	
Position, psychologist (ref)		n.s			n.s			n.s			n.s	
Managerial responsibilities		n.s			.12**	.12**		n.s			.11*	.11*

Higher standardized beta coefficients (β) indicate a stronger association; fin. β is adjusted for all previously entered variables. ^a^Total *R*^2^ = .20 , adjusted *R*^2^ = .19. ^b^Total *R*^2^ = .15, adjusted *R*^2^ = .13. ^c^Total *R*^2^ = .25, adjusted *R*^2^ = .25. ^d^Total *R*^2^ = .12, adjusted *R*^2^ = .11 * indicates significance at the *p* < .05 level, ** indicates significance at the *p* < .001 level

The model predicting the EBPAS-36 second-order component *professional concern* was statistically significant, *R*^2^ = .15, *F*(12, 730) = 10.39, *p* < .001; adjusted *R*^2^ = .13. The variables contributing significantly to the full model were as follows: years of work experience (having more years being associated with higher scores) in block 1; education (individuals with dual competence scoring lower than the reference group) in block 2; workplace (individuals in non-clinical research and educational settings and in governmental positions scoring lower, and private practitioners scoring higher than the reference groups working with outpatients), and working at a site systematically applying one or more EBI (scoring lower than individuals not working at such sites) in block 3; social and psychological work factors (the social climate being rigid/rules-based and having control over decisions scoring higher, receiving support from colleagues and experiencing empowering leadership scoring lower) in block 4; and staff role (being a non-leader higher than being a leader) in the final block. The following effects dropped out, as variables were added to the model: other continued education and being in a governmental workplace.

The model predicting EBPAS-36 second-order component *attitudes dependent on work conditions and requirements* was statistically significant (*R*^2^ = .25, *F*(9, 746) = 28.18, *p* < .0005; adjusted *R*^2^ = .25). The variables contributing significantly to the full model were as follows: age (older individuals scoring lower than younger) in block 1; workplace (combined clinical research and educational positions and private practitioners scoring lower than the reference group) in block 3; social and psychological work factors (experiencing a higher workload and a more positive social climate associated with higher scores; having control of decisions associated with lower scores) in block 4. Staff role in block 5 did not contribute statistically to the model. The following effects dropped out as variables were added to the model: gender, other continued education and working at a site systematically applying one or more EBI.

The last model predicting EBPAS-36 second-order component *attitudes related to fit and preferences* was also statistically significant (*R*^2^ = .12, *F*(7, 750) = 14.60, *p* < .0005; adjusted *R*^2^ = .11). The variables contributing significantly to the full model were as follows: gender (men scoring lower than females) in block 1, education (individuals with Ph.D. degrees scoring lower than the reference group) in block 2; social and psychological work factors (individuals experiencing more control over decisions, receiving support from colleagues and experiencing empowering leadership scoring higher); staff role (non-leaders scoring higher than leaders). The following effect dropped out as variables were added to the model: age.

## Discussion

The current article provides insights of importance for the adoption or the earliest phases of implementation processes. The first aim was to investigate the differences between professional staff roles; the second was to identify how provider demographic, social and psychological factors at work and staff roles predicted professionals’ attitudes to adopting EBI. To explore predictive factors of attitude towards adopting EBI, a simpler structure encompassing three second-order components was used following a principal component analysis of all 12 primary factors. The three components were labelled *professional concern*, *attitudes related to work conditions and requirements*, and *attitudes related to fit and preferences*. Taken together, our results suggest some group differences between staff roles. However, we also found that social and psychological organizational work factors might be more important as predictors across staff roles.

Specifically, the analyses of differences between staff roles revealed that nurses reported holding more of a positive global attitude towards adopting EBI than psychologists, while clinicians reported a more negative global attitude than non-clinicians. Possible explanations for this might include psychologists’ and nurses’ different roles and positions in a treatment setting [29]. In such settings, psychologists might have a more independent professional role than nurses and may value making independent decisions regarding which treatments to use and their delivery. Additionally, various staff roles might be more concerned with issues related to their work areas, that for the group of clinicians might be more obviously related to their frontline clinical work. This again can be seen in light of findings by for instance Green and Aarons [32] concerning different stakeholder perspectives, highlighting that issues important to clinicians are connected to the impact of interventions on the aspects of clinician practice, e.g., the therapeutic relationship and the ability to intervene to meet the needs of an individual patient. The hierarchical regression analysis demonstrated that having leader responsibility or not was a significant predictor of attitude towards EBI. Here, non-leaders attitudes were more influenced by professional concerns (i.e., the limitations of EBI, the importance of clinical experience over science, negative perceptions of monitoring, a lack of openness to new practices and the time and administrative burdens), as well as by fit and preference (i.e., the fit with the clinicians’ current approach, the perceived needs of clients and positive perceptions of feedback). In line with Stadnick et al., [31] reporting on possible variation in priorities, values and responsibilities specific to leaders and therapist, this might reflect that non-leaders feel more concern as end-users of the intervention, and hence express a greater concern with how it interferes with everyday practice.

Provider demographics and social and psychological factors at work predicted both global and second-order attitudinal components. In line with previous studies [8], we found that men reported more negative attitudes towards adopting EBI than did females. Our data provide no explanation of why males are more conservative in adopting EBI, but the finding indicate that gender in itself may be a factor that implementation efforts should be aware of. We also found that younger respondents held more positive attitudes than older respondents, on both global attitudes and *attitudes related to work conditions and requirements*, capturing issues of organizational support, as well as education, training, job security and interventions being imposed. That the younger respondents might be more occupied with issues of organizational support, training and education is consistent with previous findings by Okamura and colleagues [16], showing that younger therapists assigned greater value to job security and organizational support for learning new EBI. Younger respondents might naturally be in a period of their career where their focus is more on acquiring knowledge and skills needed for the tasks they are employed to perform, when the demands of the work environment might be more strenuous, and the necessity of the appropriate organizational support is perceived more eminent. For the sake of implementation strategies, this is not to say that organizational support and training are not important to older respondents but are perhaps more favoured by younger respondents, something which might be a valuable question for future research to address. In addition, our study showed that more years of experience also predicted a higher score on the *professional concern* domain. This emphasizes the importance of implementation strategies being tailored to the different needs and views of providers in different phases of their careers, as the more experienced individuals might be more occupied with or affected by their cumulative experiences over time and how the intervention to be implemented interferes and balances with one’s usual everyday practice. When looking at the literature on important implementation strategies, both training issues and interventions that fit with real world context have been increasingly recognised as essential to target [18, 43–45]. Developing training strategies, providing proper organizational support and securing intervention initiatives being appropriate for a given setting are not straightforward, given the complex multi-level challenges associated with implementation efforts [45, 46]. Such challenges are for instance associated with the differences between agency settings, the availability of resources, the multitude of different intervention components that are to be taught, the different needs of clinicians and the presenting problems of the clients [46]. This picture poses challenges on organizations planning intervention initiatives, for instance concerning which intervention(s) to implement and to what extent, as well the scaling of training needed [43]. It also poses both a need and encouragement for future research to continue address both which and how training efforts best can facilitate the uptake of new interventions and to secure that organizations lay grounds for learning environments fostering and motivating staff that are capable of delivering the intended service.

Regarding workplace, those working as private practitioners held more negative global attitudes and more *professional concern* compared to those working in public outpatient services, whereas those working non-clinically with research and education held more positive global attitudes and less concern regarding adoption of EBI. Being a private practitioner, as well as working in a combined position with both clinical work and research and education, also predicted a lower score on *attitudes related to work conditions and requirements*. Our ability to report the reasons for these differences, which likely also are complex, is beyond the scope of this article. However, some possible explanations which future research might consider are offered. For instance, the questions concerning work conditions and organizational support may not apply as much to those working in private practice, while questions regarding professional concern might be less familiar to those representing academia. Another possibility may be tied to the so-called science-practice gap, where private practitioners may feel more ambivalent or sceptic towards the adoption and use of EBI than those working in other settings. They may also have chosen a private direction in order to attain more autonomy than is possible within the public mental health system. Further, our results showed that employees at sites that systematically applied one EBI or more held more positive global attitudes and less *professional concerns* than not working at such sites, indicating organizational context, experience and knowledge of EBI influencing attitudes [7].

As for the social and psychological factors at work, the experience of being more in control of decisions regarding one’s own work situation predicted more professional concern, putting less value on work conditions and requirements, as well as having a greater willingness to use interventions based on shared preferences with patients and colleagues and the perception of valuing feedback from others. Additionally, empowering leadership encompassing the experience of being encouraged by superiors to participate in decision making, opinion sharing and skill development, as well as the perception of receiving collegial assistance when in need, stood out as predictors of more positive attitudes and greater willingness to use interventions based on fit and shared preferences. Experiencing an empowering leadership style and collegial assistance was also related to lower professional concern. Finally, our results supports previous research regarding barriers to adopting EBI [8, 16], with the experience of higher workloads predicting *attitudes related to work conditions and requirements*, including issues related to perceived time and administrative burden of learning new interventions, and the need of organizational support, training and education.

### Limitations

Limitations include the low response rate, a familiar problem in web-based survey studies [47] that may represent a risk of potential bias of results. As this represents less of a problem for a survey applying regression analysis than for prevalence studies, a low response rate is considered only a minor limitation of this study [48]. Another concern has to do with a potential confusion around the terms *evidence-based practice* and *evidence-based treatments and interventions*. Although they were specified in the invitation emails and written instructions, the respondents might have been confused about the actual meanings of the terms being used. Written comments also showed that certain respondents experienced difficulty providing nuanced responses to such complex concepts in a survey format. The effect size of the reported group differences between staff roles were small, hence a future study using a design that could reveal practical implications of these differences would be a natural next step. It is beyond the scope of this study to explain the differences in causal mechanisms; one should take care to avoid over-interpretation. The sole focus on the adoption or initial implementation phases is another limitation in the present study, given that later implementation phases might involve other processes and challenges of a multilevel nature [14]. The regression models predicted approximately 12–25% of the total variances. This reflects the complex mechanisms involved and that several factors in addition to those studied also contribute to attitudes towards adoption. For instance, as the Dalheim et al., study among Norwegian nurses suggested [20], lack of skills and confidence to implement EBI might act as an important barrier, regardless of level of attitudes. Future research should address these issues using a variety of research methodologies to obtain a deeper and more comprehensive understanding of the complexities of implementation challenges.

## Conclusion

Implementation efforts in healthcare service settings are particularly prone to challenges, as they are dependent on both the actions of every individual practitioner as well as organizational influences within the complex and constantly changing contexts of a hospital or healthcare delivery environment [14]. Previous studies have both observed and failed to observe discrepancies between different staff roles and professions concerning attitudes towards adopting EBI [19, 28, 29]. However*,* mental health providers constitute a heterogeneous group of individuals with various backgrounds, roles and disciplines that might impact their views on adopting evidence-based interventions. Taken together, our findings highlight the importance of both individual and organizational factors by indicating that professionals’ attitudes towards change and innovation are influenced by the inner context of their work environment, being the supportiveness by colleagues and the leadership style of superiors, as well as individual autonomy, years of experience, work conditions and organizational characteristics. Solutions to barriers need to be directed to the dimension where the barrier occurs while recognizing that multilevel approaches are essential to success in overcoming barriers [15, 49, 50]. To ignore both the knowledge of and potential causes of providers’ concerns about using EBI might be quite risky. First, ignoring the viewpoints of clinicians may widen the so-called scientist-practitioner gap. Furthermore, ignoring clinicians’ concerns and their overall work environment may hamper the substantial efforts applied to implement interventions in the real-world practice settings, leading to waste of invested resources. Our results suggest that proper training, adequate organizational support, a working environment accepting professionals’ influence on important decisions related to adoption of new interventions, experiencing colleagues’ support and an empowering leadership style, may encourage the experience of participation in implementation processes, as well as opportunities for personal development. As organizational leadership plays an important role in improving the context for adoption of interventions [7, 25], mental health service organizations may benefit from improving leadership skills in preparation for implementing EBI, thereby establishing a foundation for a work and collegial climate where learning and new skills development might flourish. In such an environment, different staff roles and positions have different level of experience, perspectives, needs and values that are important to them. This again has implications for the choice and design of training and support efforts that most efficiently lead to successful adoption and sustainability of EBI, with the ultimate goal of advances in psychotherapy research reaching the actual people in need.

## Abbreviations

CFA: Confirmatory factor analysis
EBI: Evidence-based interventions
EBP: Evidence-based practice
EBPAS: The Evidence-based Practice Attitude Scale
PCA: Principal component analysis
